# Seed morphometric characteristics of European species of *Elatine* (Elatinaceae)

**DOI:** 10.7717/peerj.3399

**Published:** 2017-05-31

**Authors:** Agnieszka Popiela, Andrzej Łysko, Bożenna Białecka, Magdalena Marta Bihun, Gábor Sramkó, Waldemar Staroń, Anetta Wieczorek, Attila Molnár V.

**Affiliations:** 1 Department of Botany and Nature Conservation, University of Szczecin, Szczecin, Poland; 2 Department of Environmental Protection and Management, Western Pomeranian University of Technology in Szczecin, Szczecin, Poland; 3 Molecular Biology and Biotechnology Center, University of Szczecin, Szczecin, Poland; 4 Department of Botany, MTA-DE ‘Lendület’ Evolutionary Phylogenomics Research Group, University of Debrecen, Debrecen, Hungary; 5 Institute of Physics, University of Szczecin, Szczecin, Poland; 6 Department of Ecology, University of Szczecin, Szczecin, Poland; 7 Department of Botany, University of Debrecen, Debrecen, Hungary

**Keywords:** Determination key, Ephemerals, Amphibious species, Seed coat, Morphology, Malphigiales, SEM, Micromorphology, Seeds traits, Population

## Abstract

*Elatine* L. contains ca. 25 small, herbaceous, annual species distributed in ephemeral waters in both hemispheres. All species are amphibious and characterized by a high degree of morphological variability. The importance of seed morphology in *Elatine* taxonomy has been emphasized by many authors. The degree of seed curvature and seed coat reticulation have been traditionally considered very important in recognizing individual species of this genus. Seed morphometric characteristics of 10 *Elatine* species, including all European native taxa, are provided on the basis of material from two or three populations of each species. A total of 24–50 seeds were studied from each population, altogether 1,260 images were used for the morphometric study. In total, six parameters were measured from SEM pictures: object surface area, profile specific perimeter (object circuit), rectangle of the object (a) length, rectangle of the object (b) width, angle of the seed curvature, and number of pits in the seed coat counted in the middle row. Our study shows that the range of morphological variation of seeds in European species of *Elatine* is great, both between the species and the populations. Discrimination analysis showed that all six traits significantly differentiate the populations studied (*λ* = 0.001, *p* < 0.001), and the greatest contributions were “number of pits”, “rectangle_a”, and “the angle curvature”. Multidimensional scaling based on a correlation matrix of Mahalanobis distance of the six features studied revealed the greatest similarity between the three populations of *E. alsinastrum, E. macropoda,* and* E. hexandra*. Regarding interspecific differences, a Kruskal–Wallis tests showed that, in many cases, lack of statistically significant differences between species relative to the studied seed traits. If distinction of species is only based on seeds, especially if only a few seeds are evaluated, the following species pairs can be easily confused: *E. alsinastrum* and *E. orthosperma*, *E. hexandra* and *E. macropoda*, *E. campylosperma* and* E. hydropiper,* as well and *E. gussonei* and *E. hungarica*. We found no diversity in seed coat micromorphology within pits that could have potential taxonomic importance. An identification key and descriptions of species are provided on the basis of seeds traits.

## Introduction

*Elatine* L. is one of the two genera in the Elatinaceae, a family in Malpighiales (Tucker, 1986; Davis & Chase, 2004), and contains ca. 15–25 ephemeral amphibious species (Heywood et al., 2007). To the present knowledge, ten native taxa occur in Europe; however, Flora Europea lists seven (Cook, 1968) and Euro+Med Plantbase nine native species (Uotila, 2009b). One taxon belongs to the subgenus *Potamopithys* (Adanson) Seub(*E. alsinastrum* L.), and the other taxa are classified into subgenus *Elatine* Seub. (=*Hydropiper* Moesz): *E. triandra* Schkuhr (sect. *Triandra* Seub. (=*Crypta* (Nutt.) Seub.); *E. brochonii* Clavaud, *E. campylosperma* Seub., *E. gussonei* (Sommier) Brullo, Lanfr., Pavone & Ronsisv., *E. hexandra* (Lapierre) DC., *E. hydropiper* L., *E. hungarica* Moesz, *E. macropoda* Guss., and *E. orthosperma* Düben (section: *Elatinella* Seub.). Two more taxa of sect. *Elatinella* occur in the New World (in North America *E. californica* A. Gray and in South American *E. ecuadoriensis* Molau). Other taxa classified to sect. *Triandra* mainly occur in temperate regions of the Old and New World, with the probable center of diversity in North and South America. *E. ambigua* is another taxon from Europe (Uotila, 2009b). It shows no substantial genetic differences in relation to *E. triandra* (Sramkó et al., 2016) an Asian species also occurring in Europe.

*Elatine alsinastrum* is characterized by whorled leaves; all other species have opposite leaves, and are mainly distinguished by number of stamens (three, six or eight) and number of perianth lobes (three or four). The shape of leaves is variable, oblong or roundish, petiolate or almost sessile, and depends on environmental conditions. Flowers are sessile or pedunculated, while tiny seeds are oblong, curved or horseshoe-shaped (Cook, 1968; Tucker, 1986).

Recently, *Elatine* species have been of interest to researchers because of their rarity throughout their range, relatively poorly known distribution and taxonomy, ecology, karyology and phylogenetic relationships (e.g.,  Popiela, 2005; Misfud, 2006; Uotila, 2009a; Uotila, 2010; Popiela & Łysko, 2010; Popiela & Łysko, 2011; Popiela et al., 2011; Popiela et al., 2012; Popiela, Łysko & Molnár, 2013; Popiela et al., 2015; Takács, 2013; Molnár et al., 2013; Molnár, Popiela & Lukács, 2013; Šumberova & Hrivnak, 2013; Kalinka et al., 2014; Kalinka et al., 2015; Cai et al., 2016; Sramkó et al., 2016). The above-mentioned authors emphasized that the erratic temporal appearance of *Elatine* species depends mainly on environmental factors; for example, plants develop as aquatic or terrestrial forms, and, moreover, they are morphologically variable depending on the phase of drying on the ground. This variability and the very small size of plants and short-lasting, tiny flowers often make proper identifications difficult. Earlier leaf length and shape, pedicel length and seed shape were widely used for identification of *Elatine* taxa (Seubert, 1845; Niedenzu, 1925). The importance of seed morphology in *Elatine* taxonomy has been emphasized by many authors: the degree of seed curvature (i.e., seed shape) and seed coat reticulation have been considered very importan tfor recognizing individual species (Cook, 1968; Uotila, 1974; Uotila, 2010; Tucker, 1986; Misfud, 2006; Molnár et al., 2013).

There have been only a few studies addressing morphological variability of *Elatine* taxa (Mason, 1956; Molnár et al., 2013; Molnár, Popiela & Lukács, 2013). Recently, (Molnár et al., 2015) examined the level of phenotypic plasticity in *Elatine.* Analysis of morphological differences between aquatic and terrestrial forms of individual species clearly showed that vegetative traits are highly influenced by environmental factors and only seed traits are stable within species. According to Molnár et al. (2015), only seed morphology (aside from generative characteristics) is valuable for taxonomic purposes.

Consequently, we studied seed morphometric characteristics of 10 *Elatine* species, including all native European taxa, as a part of comprehensive surveys on taxonomy and phytogeography of this genus that have been conducted by a Hungarian-Polish research team since 2010. We assumed that advanced and methodically uniform seed characteristics are taxonomically important in this genus. Our aims were to (i) find statistical differences between *Elatine* species relative to seed morphological features, (ii) evaluate intra- and interspeciesseed variability, and then (iii) construct a guide to identifying species based onseed morphological features. Due to the small size of seeds the study was made by using SEM micrographs.

## Material & Methods

### Plant material and cultivation

**Figure 1:**
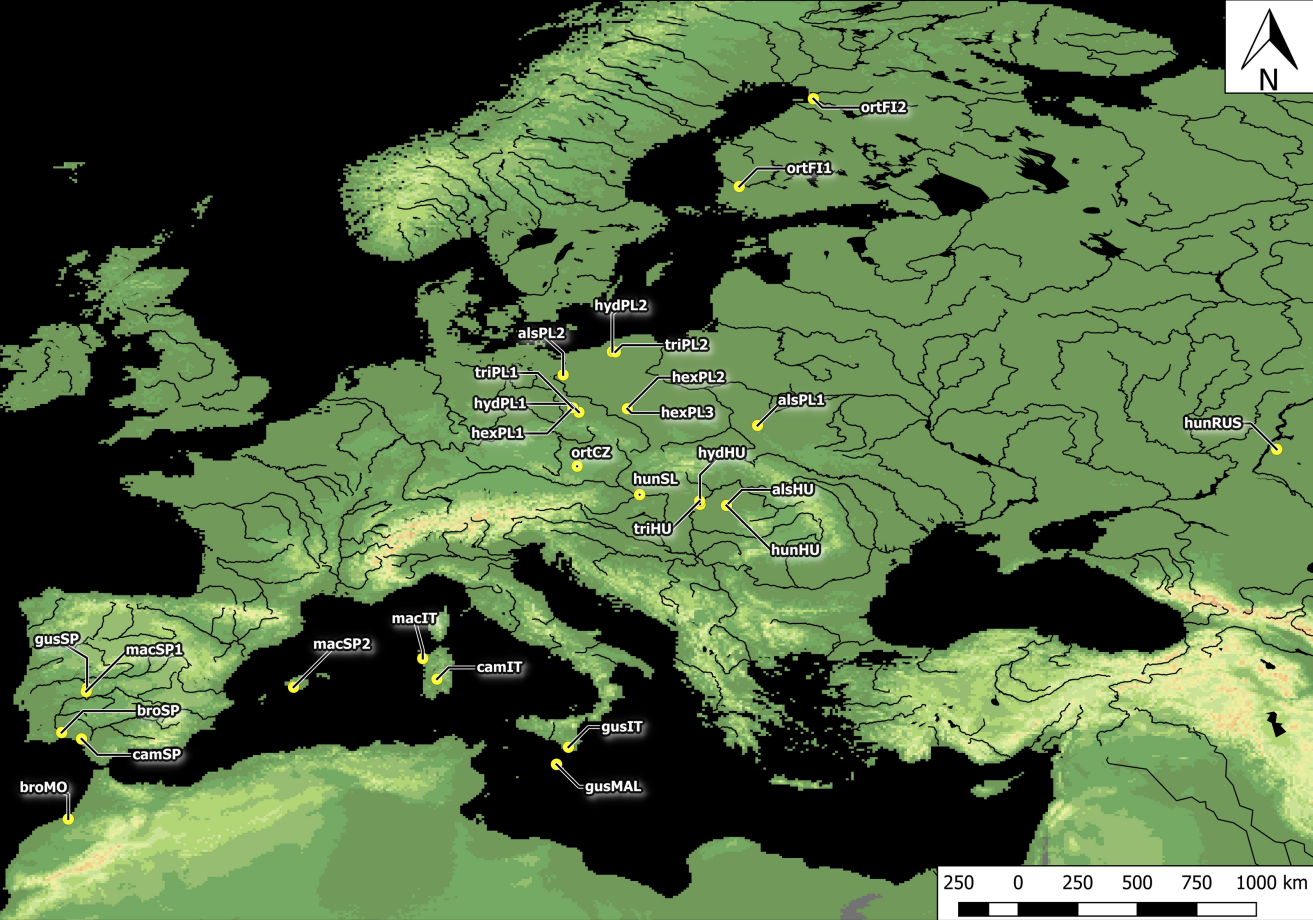
Distribution of *Elatine* populations studied. For acronyms, see Table 1.

Plants studied were collected across Europe. In total, seeds were collected from all 10 *Elatine* species and from three populations each, with an exception of very rare *E. brochonii* and *E. campylosperma,* two populations, so altogether 28 populations were used for the study. The distance between the populations of each species ranged from approximately 10–2,000 km. For the localities of the original material, and the voucher specimens, see Table 1 and Fig. 1. The studied seeds were gathered directly from the field, or from cultivated plants grown from the original material; in some cases seeds from herbarium specimens were used. Culture was conducted at the Center for Molecular Biology at the University of Szczecin, Poland and/or a the Department of Botany at the University of Debrecen, Hungary. Plants were grown in climate-controlled culture chambers with 12 h/day light and 30,000 lux light intensity, temperatures: under light, 22 ± 2 °C, and under dark, 18 ± 2 °C.

**Table 1:** The species of *Elatine* and their populations included in the study, with the acronyms of the populations used in text, figs and tables.

Nr	Acronym	Name	Origin^*^^,^^**^	Latitude	Longitude	Collector, voucher	No. of seeds	Approx. distance between two/three populations (km)
1.	alsHU	*E. alsinastrum* L.	Hungary: Konyár^**^	47.31	21.67	*Molnár V. A.* DE*-* 22226	50	620
2.	alsPL1	*E. alsinastrum* L.	Poland: Staw Noakowski^*^	50.80	23.03	*Popiela A*. SZUB*-* 008756	50	
3.	alsPL2	*E. alsinastrum* L.	Poland: Strzelczyn	53.01	14.54	*Popiela A.* SZUB*-* 015968	50	
4.	broMO	*E. brochonii* Clavaud	Morocco: Ben Slimane^**^	33.62	−7.07	*Lukács B. A. DE-43230*	44	420
5.	broSP	*E. brochonii* Clavaud	Spain: San Silvestre de Guzmán^**^	37.4	−7.36	*Molnár V. A. DE-37684*	49	
6.	camIT	*E. campylosperma* Seub.	Italy: Sardegna, Gesturi^**^	39.73	9.03	*Molnár V. A. DE-37423*	47	1,380
7.	camSP	*E. campylosperma* Seub.	Spain: El Rocio, Donana^**^	37.12	−6.49	*Molnár V. A. DE-37681*	50	
8.	gusMAL	*E. gussonei* (Sommier) Brullo, Lanfr., Pavone & Ronsisv.	Malta: Gózó: Ta’ Sannat^**^	36.01	14.25	*Molnár V. A. & Lukács B. A. DE-43229*	50	1,265
9.	gusSP	*E. gussonei* (Sommier) Brullo, Lanfr. Pavone & Ronsisv.	Spain: Casar de Cáceres^**^	39.33	−6.25	*Molnár V. A. DE-43231*	50	
10.	gusIT	*E. gussonei*	Italy: Sicily, Modica^**^	36.76	14.77	*Molnár V. A. DE-38750*	50	
11.	hexPL1	*E. hexandra* (Lapierre)DC.	Poland: Janików (Janikowo)	51.57	14.96	*Popiela A.* SZUB*-* 015964	33	115
12.	hexPL2	*E. hexandra* (Lapierre) DC.	Poland: Milicz^*^	51.55	17.35	*Popiela A.* SZUB*-* 010851	50	
13.	hexPL3	*E. hexandra* (Lapierre) DC.	Poland: Ruda Milicka^*^	51.53	17.34	*Dajdok Z.* SZUB*-* 011097	50	
14.	hunRUS	*E. hungarica* Moesz	Russia: Volgograd^**^	49.76	45.7	*Mesterházy A. DE-37484*	42	1,375
15.	hunSLO	*E. hungarica* Moesz	Slovakia: Okánikowo	47.78	17.88	*Eliáš P*. SZUB*-* 010523	*24*	
16.	hunHU	*E. hungarica* Moesz	Hungary: Konyár^**^	47.31	21.67	*Molnár V. A. DE-22266*	50	
17.	hydHU	*E. hydropiper* L.	Hungary: Tiszagyenda^**^	47.36	20.52	*Molnár V. A. DE-22273*	39	550
18.	hydPL1	*E. hydropiper* L.	Poland: Parowa	51.38	15.23	*Popiela A.*	45	
19.	hydPL2	*E. hydropiper* L.	Poland: Kwecko Lake	54.02	16.69	*Popiela A.,Prajs B.* SZUB*-* 015705	43	
20.	macIT	*E. macropoda* Guss.	Italy: Sardegna: Olmedo^**^	40.63	8.41	*Molnár V. A. DE-37424*	50	900
21.	macSP1	*E. macropoda* Guss.	Spain: Casar de Cáceres^**^	39.19	−6.29	*Molnár V. A. DE-37692*	46	
22.	macSP2	*E. macropoda* Guss.	Spain: Mallorca: Cap Blanc/SaTore^*^	39.38	2.77	*Popiela A.*, SZUB”-*-* 015969	42	
23.	ortCZ	*E. orthosperma* Düben	Czech Republic: Klášter^*^	49.02	15.15	*Šumberova K.*	45	1,260
24.	ortFI1	*E. orthosperma* Düben	Finland: Kokemäki	61.23	22.23	*Suominen J,* H 439800	25	
25.	ortFI2	*E. orthosperma* Düben	Finland: Oulu^*^	65.06	25.47	*Mesterházy A. DE-43232*	50	
26.	triHU	*E. triandra* Schkuhr	Hungary: Kisköre^*^	47.50	20.50	*Molnár V. A. DE-22282*	41	570
27.	triPL1	*E. triandra* Schkuhr	Poland: Janików	51.57	14.96	*Popiela A*., SZUB*-* 010520	47	
28.	triPL2	*E. triandra* Schkuhr	Poland: Bobięcińskie Małe Lake	54.01	16.82	*Dambska I.,* SZUB*-* 010862	50	

**Notes.**

* cultivation in Poland

** cultivation in Hungary

*Elatine hungarica*, *E. hydropiper* and *E. triandra* are protected species in Hungary and were sampled with the permission of the Hortobágy National Park Directorate (Permission id.: 45-2/2000, 250-2/2001).

**Figure 2:**
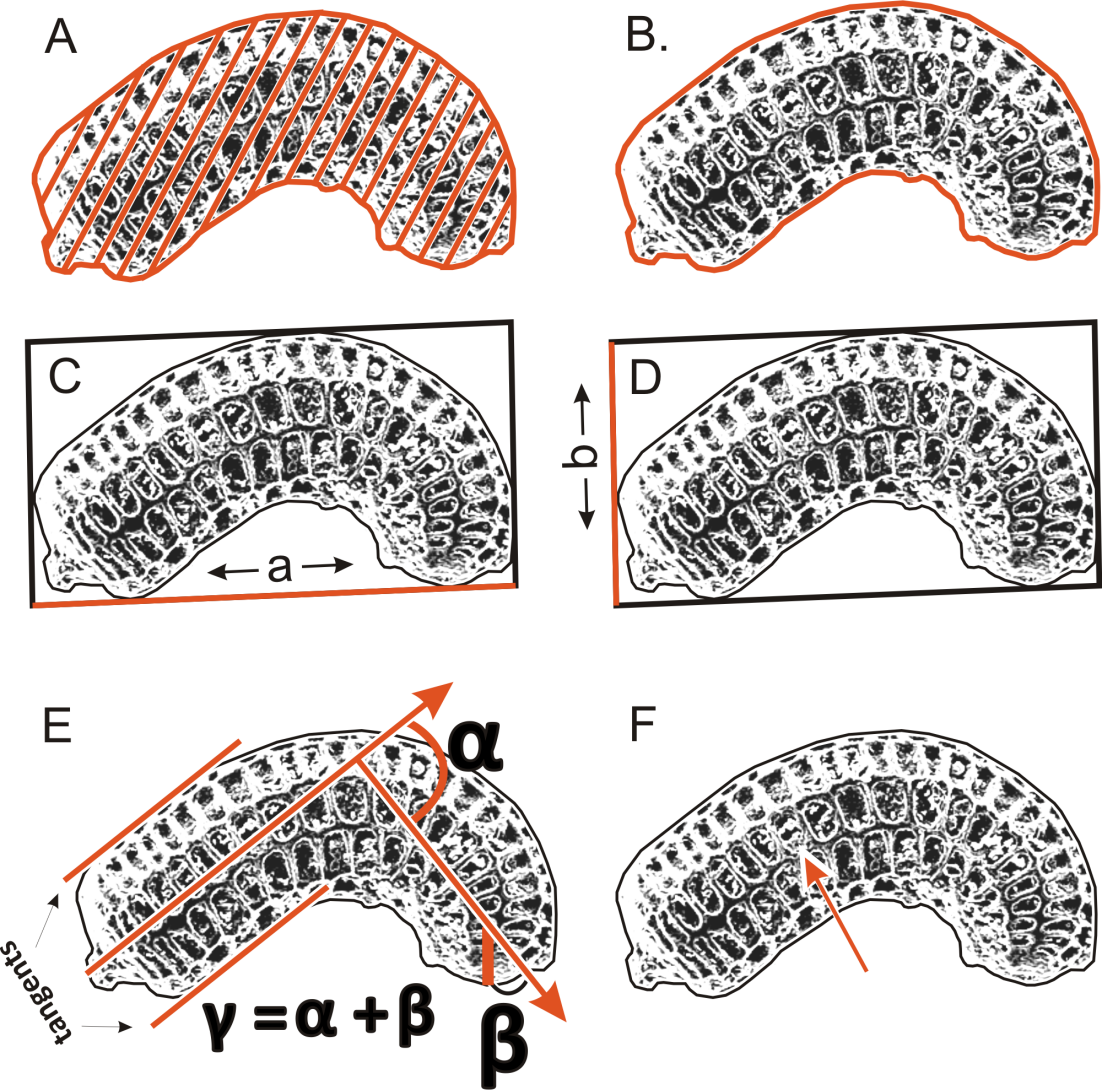
The method of measuring of seed. (A) surface; (B) profile; (C) rectangle a; (D) rectangle b; (E) the angle of curvature (*γ* = *α* + *β*); (F) number of pits in the middle row.

To determinate the variability and diagnostic features of seeds, 24–50 seeds obtained from several individuals from each population (Table 1) were used. A total of more than 1,500 scanning electron microscope (SEM) images of the seeds were obtained at ×200 magnification using an SEM (Zeiss Evo, Molecular Biology and Biotechnology Center, University of Szczecin, Szczecin, Poland); however, 1,260 images were used for the morphometric study, because all cracked seeds were excluded. In total, six parameters were measured (Fig. 2): (A) object surface area; (B) profile specific perimeter (object circuit); (C) object rectangle a (length); (D) object rectangle b (width); (E) the angle of curvature; (F) number of pits on the seed coat counted in the middle row. Moreover, membrane presence and pit shape were evaluated.

To examine micromorphology of seed coat, 48 pictures at ×500, ×2,000, ×4,000, and ×7,000 were taken (Zeiss Evo SEM; Laboratory of Confocal and Electron Microscopy, Faculty of Biology, Adam Mickiewicz University, Poznań, Poland)

### Data analysis

To distinguish the characteristics that have the greatest impact on population and species discrimination, multiple discriminant analysis was used. Wilks *λ* was used to measure the discriminatory power of the model (0—perfect discrimination; 1—no discrimination). Interpretation of discriminant functions was performed using canonical analysis.

For visualization of the relationship between species and populations, Mahalanobis distance-based unweighted pair-group method using arithmetic averages to construct (UPGMA) trees was applied. Canonical values were shown using categorized scatterplots. The most discriminative traits were also independently tested by the non-parametric Kruskal–Wallis tests. All calculations were made in Statistica v. 12.5 software.

## Results

### Variability of populations within species

Discrimination analysis showed that all six traits significantly differentiate the populations studied (*λ* = 0.001, *p* < 0.001). Of these, the greatest contributions were as follows: number of pits, rectangle a (length), and the angle of curvature (Table 2).

Based on a Kruskal–Wallis tests, we found no statistically significant differences (*p* = 0.05) between populations studied of each European *Elatine* species regarding the following traits: (A) surface (all species except *E. campylosperma, E. hungarica, E. hydropiper*), (B) profile (all species except *E. campylosperma, E. hexandra, E. hungarica, E. hydropiper*), (C) rectangle a (all species except *E. brochonii*), (D) rectangle b (all species), (E) the angle of curvature (all species), (F) number of pits (all species except *E. gussonei, E. hungarica, E. triandra*) (Table 3). Accordingly, the traits studied did not show statistically significant variation between populationsof the following species: *E. alsinastrum, E. macropoda* and *E. orthosperma.*

**Table 2:** Discriminant analysis of the studied populations of *Elatine*.

*N* = 1,260	*λ* = .00016*F*(162, 7217) = 153.07*p* < 0.0001					
	*λ* (Wilks)	Fragm. (Wilks)	F (27.1227)	*p*	Toler.	*R*^2^
The angle of curvature	0.000461	0.342896	**87.0870**	0.0001	0.751829	0.248171
Rectangle a	0.000492	0.320751	**96.2370**	0.0001	0.254119	0.745881
Number of pits	0.000839	0.188320	**195.8703**	0.0001	0.989553	0.010447
Surface	0.000298	0.530541	40.2124	0.0001	0.169730	0.830270
Rectangle b	0.000229	0.690923	20.3291	0.0001	0.262387	0.737613
Profile	0.000199	0.793047	11.8592	0.0001	0.632832	0.367169

**Figure 3:**
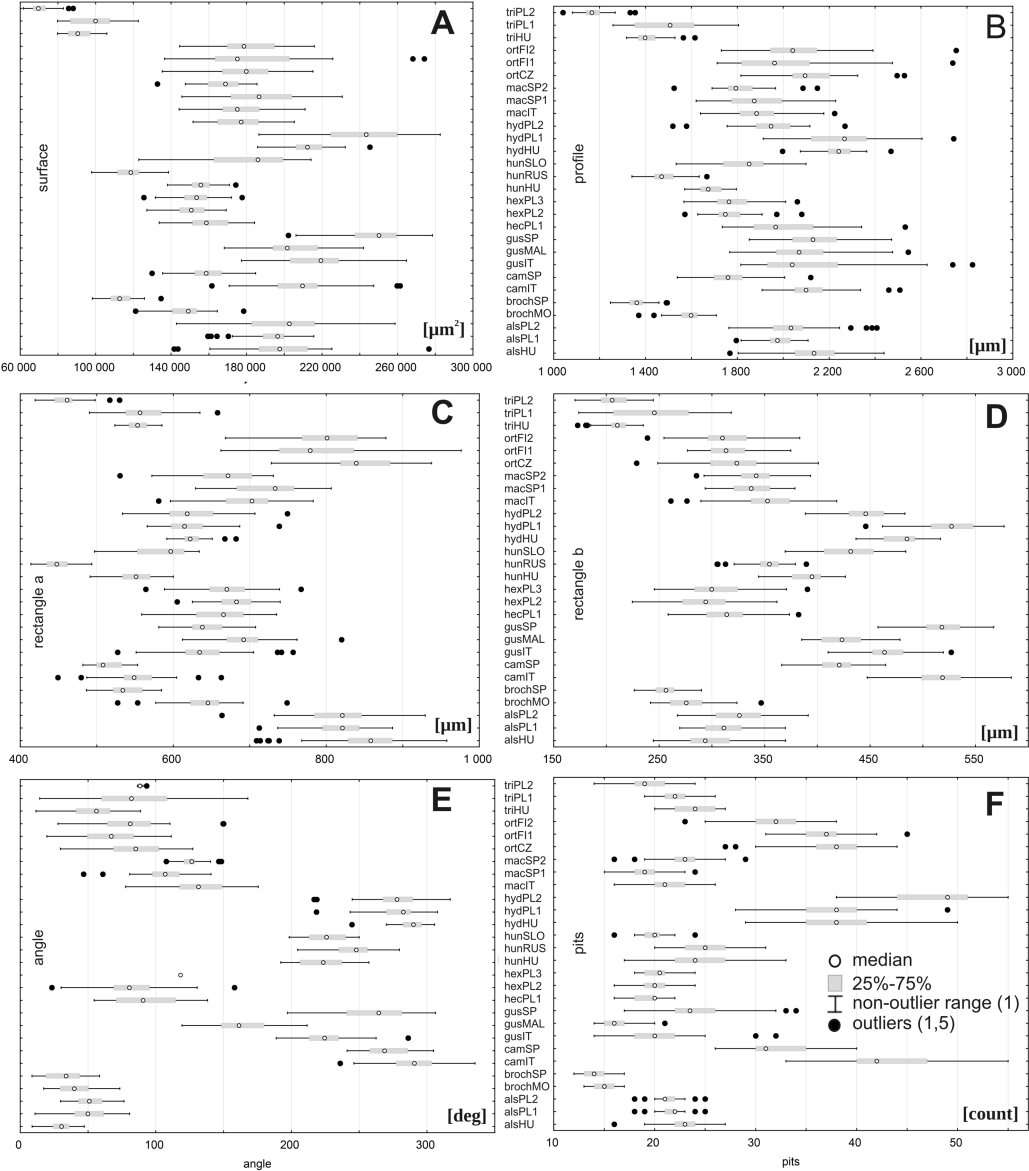
Boxplots of the most discriminative seed traits among 28 studied populations of *Elatine*. Notations: boxes indicate 25–75 percentiles, white point indicate medians, whiskers exclude outliers, black points indicate outliers. For acronyms, see Table 1. (A) surface; (B) profile; (C) rectangle a; (D) rectangle b; (E) angle; (F) pits.

However, there were large ranges of variation for some traits, especially within the following populations: *E. orthosperma* from Finland, Fin1 (for acronyms see Table 1) (surface: SD 36131.9 and rectangle a: SD 78.9), *E. hungarica* from Slovakia (profile: SD 498.1), *E. triandra* from Poland, PL1 (rectangle b:SD 39.9 and the angle of curvature: SD 33.7), and *E. hydropiper* from Hungary (number of pits: SD 5.3) (Fig. 3, Table 4). Conversely, the smallest ranges of variation were observed in the following populations: *E. triandra* from Poland, PL2 (surface: SD 5897.8), *E. triandra* from Hungary (rectangle a: SD 15.3), *E. brochonii* from Spain (profile: SD 51.7 and rectangle b: SD 13.6), *E. hydropiper* from Hungary and *E. macropoda* from Spain, SP2 (angle of curvature, SD 10.5; SD 10.3, respectively), and *E. brochonii* from Morocco (number of pits: SD 0.9) (Fig. 3, Table 4).

**Figure 4:**
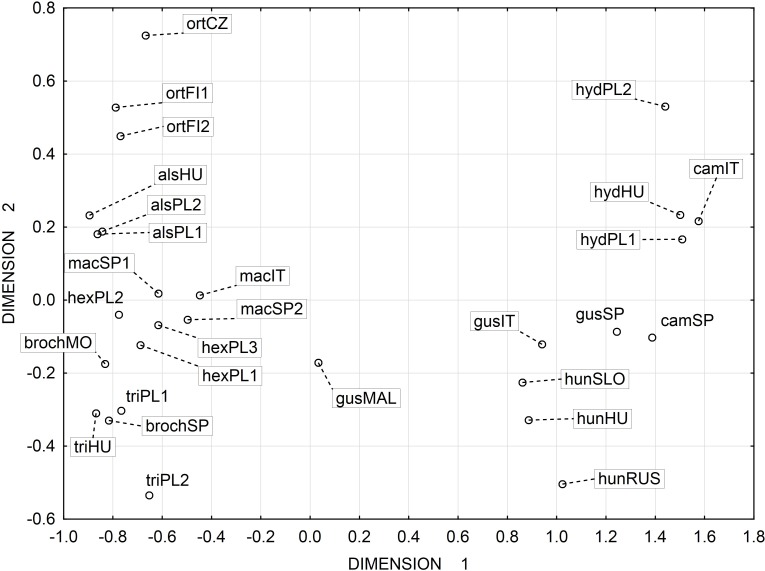
Multidimensional scaling based on a correlation matrix of Mahalanobis distance for seed traits among 28 populations of *Elatine.* For acronyms, see Table 1

Multidimensional scaling based on a correlation matrix of Mahalanobis distance of the six features studied revealed the greatest similarity between the three populations of the following species: *E. alsinastrum, E. macropoda, E. hexandra* (Fig. 4).

### Variability between species

Discriminant analysis showed that all variables could discriminate species (*λ* < 0.01). The greatest impact was from the following features: number of pits, the angle of curvature and rectangle a (Table 5).

**Table 3:** Significant differences between populations of *Elatine.* based on Kruskal–Wallis tests (*p* = 0.05). For acronyms, see Table 1.

	alsHU	alsPL1	alsPL2	brochMO	brochSP	camIT	camSP	gusIT	gusMAL	gusSP	hexPL1	hexPL2	hexPL3	hunHU	hunRUS	hunSLO	hydHU	hydPL1	hydPL2	macIT	macSP1	macSP2	ortCZ	ortFI1	ortFI2	triHU	triPL1	triPL2
alsHU				abcf	abcf	cdef	abcdef	cde	cdef	acde	acef	abce	abce	abcde	abcde	bcde	cdef	cdef	cdef	bcde	bef	abce	ef	f	ef	abcd	abce	abcdef
alsPL1				abcf	abcdf	cdef	abcdef	cde	cdef	acde	ac	abc	abce	abcde	abce	cde	bcdef	abcdef	cdef	ce	e	ce	f	f	f	abcd	abc	abcd
alsPL2				abcf	abcdf	cdef	abcdef	cde	cdef	acde	ac	abc	abce	abcde	abce	cde	cdef	cdef	cdef	ce	e	abce	f	f	f	abcd	abcd	abcd
brochMO	abcf	abcf	abcf		c	abcdef	cdef	abdef	abde	abdef	be	f	ef	cdef	cdef	de	abdef	abdef	abdef	abdef	abcde	def	abcf	abcf	abcf	cf	f	ace
brochSP	abcf	abcdf	abcdf	c		abdef	abdef	abcdef	abcde	abcdef	abce	bcef	bcef	def	def	abde	abcdef	abcdef	abcdef	abcdef	abcdef	abcdef	abcdef	abcf	abcdef	f	ef	ef
camIT	cdef	cdef	cdef	abcdef	abdef		ab	cf	cef	cf	acdef	abcdef	abcdef	abdf	abdf	bf			a	abcdef	bcdef	abcdef	cde	cde	cde	abdef	abdef	abdef
camSP	abcdef	abcdef	abcdef	cdef	abdef	ab		abcf	abcef	abcf	bcdef	cdef	cdef	f		f	abc	abc	c	cef	acdef	cdef	bcde	cde	bcde	abde	adef	abdef
gusIT	cde	cde	cde	abdef	abcdef	cf	abcf			f	ade	abde	abd	abcf	abcdf		f	f	af	ad	cde	abd	acdef	cdef	acdef	abcdef	abcde	abcde
gusMAL	cdef	cdef	cdef	abde	abcde	cef	abcef			f	ad	abde	abdf	abcf	abcf	c	ef	ef	ef	f	d	abdf	cdef	def	def	abcdef	abcdef	abcde
gusSP	acde	acde	acde	abdef	abcdef	cf	abcf	f	f		adef	abdef	abde	abcd	abcd	ab	f	f	af	abde	abcdef	abde	acdef	acdef	acdef	abcde	abcde	abcdef
hexPL1	acef	ac	ac	be	abce	acdef	bcdef	ade	ad	adef		b	b	bcdef	bcef	de	adef	adef	def				cf	f	cf	abcdf	abc	abcd
hexPL2	abce	abc	abc	f	bcef	abcdef	cdef	abde	abde	abdef	b			cdef	cdef	cde	abdef	abdef	adef	ade	a		abcf	f	abcf	bcf	c	abcd
hexPL3	abce	abce	abce	ef	bcef	abcdef	cdef	abd	abdf	abde	b			cd	bcef	cd	abdef	abdef	def		a		abcf	f	abcf	abcdef	abc	abcd
hunHU	abcde	abcde	abcde	cdef	def	abdf	f	abcf	abcf	abcd	bcdef	cdef	cd				abf	abdf	bf	bc	abcef	c	bcdef	bcdef	bcdef	ade	ade	abdef
hunRUS	abcde	abce	abce	cdef	def	abdf		abcdf	abcf	abcd	bcef	cdef	bcef			abf	abcdf	abcdf	abcf	abc	abcef	abc	abcef	abce	abce	de	de	def
hunSLO	bcde	cde	cde	de	abde	bf	f		c	ab	de	cde	cd		abf		bf	abf	f	c	c		bcdef	cdef	cdef	abde	abde	abde
hydHU	cdef	bcdef	cdef	abdef	abcdef		abc	f	ef	f	adef	abdef	abdef	abf	abcdf	bf			ab	abdef	bcdef	abdef	acde	cde	acde	abdef	abdef	abcdef
hydPL1	cdef	abcdef	cdef	abdef	abcdef		abc	f	ef	f	adef	abdef	abdef	abdf	abcdf	abf			ab	abcdef	abcdef	abdef	acde	acde	acde	abdef	abdef	abcdef
hydPL2	cdef	cdef	cdef	abdef	abcdef	a	c	af	ef	af	def	adef	def	bf	abcf	f	ab	ab		def	cdef	def	cde	cde	cde	abdef	abdef	abcdef
macIT	bcde	ce	ce	abdef	abcdef	abcdef	cef	ad	f	abde		ade		bc	abc	c	abdef	abcdef	def				bcf	ef	ef	abcde	abcd	abcd
macSP1	bef	e	e	abcde	abcdef	bcdef	acdef	cde	d	abcdef		a	a	abcef	abcef	c	bcdef	abcdef	cdef				bf	f	f	abcdef	abcd	abcd
macSP2	abce	ce	abce	def	abcdef	abcdef	cdef	abd	abdf	abde				c	abc		abdef	abdef	def				bcf	ef	bcf	abcde	abcd	abcdf
ortCZ	ef	f	f	abcf	abcdef	cde	bcde	acdef	cdef	acdef	cf	abcf	abcf	bcdef	abcef	bcdef	acde	acde	cde	bcf	bf	bcf				abcdf	abcdf	abcdf
ortFI1	f	f	f	abcf	abcf	cde	cde	cdef	def	acdef	f	f	f	bcdef	abce	cdef	cde	acde	cde	ef	f	ef				abcd	abcf	abcdf
ortFI2	ef	f	f	abcf	abcdef	cde	bcde	acdef	def	acdef	cf	abcf	abcf	bcdef	abce	cdef	acde	acde	cde	ef	f	bcf				abcd	abcdf	abcdf
triHU	abcd	abcd	abcd	cf	f	abdef	abde	abcdef	abcdef	abcde	abcdf	bcf	abcdef	ade	de	abde	abdef	abdef	abdef	abcde	abcdef	abcde	abcdf	abcd	abcd			f
triPL1	abce	abc	abcd	f	ef	abdef	adef	abcde	abcdef	abcde	abc	c	abc	ade	de	abde	abdef	abdef	abdef	abcd	abcd	abcd	abcdf	abcf	abcdf			
triPL2	abcdef	abcd	abcd	ace	ef	abdef	abdef	abcde	abcde	abcdef	abcd	abcd	abcd	abdef	def	abde	abcdef	abcdef	abcdef	abcd	abcd	abcdf	abcdf	abcdf	abcdf	f		

**Notes.**

a surface b profile c rectangle a d rectangle b e angle of curvature f number of pits

**Table 4:** SD values of seed traits studied of the populations of *Elatine*. For acronyms, see Table 1.

Acronym	Surface (µm^2^)	Profile (µm)	Rectangle_a (µm)	Rectangle_b (µm)	The angle of curvature (°)	Numer of pits
alsHU	22108.8	152.4	55.0	26.1	12.5	2.1
alsPL1	12280.7	69.8	34.2	22.7	16.8	1.3
alsPL2	23478.5	139.6	51.6	28.0	11.7	1.5
brochMO	14826.5	86.4	37.3	21.0	14.2	0.9
brochSP	7424.4	51.7	22.7	13.6	14.2	1.3
camIT	23754.8	154.9	37.4	34.7	21.4	5.0
camSP	12567.2	106.3	19.7	23.1	16.1	3.6
gusIT	23082.4	237.0	44.7	27.4	17.7	3.4
gusMAL	18526.5	172.1	39.8	24.8	23.0	1.6
gusSP	17567.0	121.0	29.1	26.0	26.9	4.1
hexPL1	12783.1	194.6	41.8	27.8	26.1	1.6
hexPL2	9186.5	87.2	27.3	26.0	23.6	1.7
hexPL3	10467.0	110.1	39.3	32.9	10.3	1.4
hunHU	7222.9	59.2	24.9	19.4	17.4	4.0
hunRUS	8318.2	84.2	17.5	17.8	17.4	2.7
hunSLO	24936.3	498.1	40.1	30.3	15.9	1.9
hydHU	11886.9	101.5	18.3	19.2	10.5	5.3
hydPL1	22799.7	252.3	35.7	30.5	16.7	4.2
hydPL2	12877.0	221.3	41.1	22.2	20.6	4.3
macIT	14752.8	121.9	45.0	30.6	21.4	2.1
macSP1	21061.9	153.9	45.6	22.4	18.3	1.8
macSP2	16799.6	160.0	41.1	35.2	10.3	2.5
ortCZ	18174.3	271.2	51.0	34.8	25.7	3.8
ortFI1	36131.9	241.2	78.9	24.5	24.3	3.0
ortFI2	17093.8	177.3	45.3	30.7	25.4	3.0
triHU	6365.6	67.3	15.3	13.9	16.4	2.0
triPL1	11403.4	140.7	36.4	39.9	33.7	1.8
triPL2	5897.8	55.6	22.1	18.5	11.7	2.0

**Table 5:** Discriminant analysis of seed traits studied of the European *Elatine* species.

*N* = 1,260	*λ*Wilks: .00252 *F* (54,6352) = 262.68 *p* < 0.0001					
	*λ* (Wilks)	Fragm. (Wilks)	*F* (9.1245)	*p*	Toler.	*R*^2^
The angle of curvature	0.005051	0.499212	**138.7702**	0.0001	0.632234	0.367766
Rectangle a	0.004048	0.622838	**83.7685**	0.0001	0.298593	0.701407
Number of pits	0.007668	0.328805	**282.3820**	0.0001	0.916368	0.083632
Surface	0.002735	0.921836	11.7295	0.0001	0.153021	0.846979
Profile	0.002759	0.913793	13.0504	0.0001	0.186761	0.813239
Rectangle b	0.002717	0.927840	10.7585	0.0001	0.509627	0.490373

The Kruskal–Wallis tests showed, in many cases, lack of statistical significance between species relative to the studied seed traits (Table 6). Regarding the trait surface, only *E. triandra* seeds showed statistical significance compared with all species tested. Analysis of all characteristics showed the least amount of statistically significant differences between the following species pairs: *E. alsinastrum* and *E. orthosperma*, *E. hexandra* and *E. macropoda,* as well as *E. gussonei* and *E. hydropiper* (Table 6).

**Table 6:** Significant differences of studied features between the European species of *Elatine* based on Kruskal–Wallis tests ( *p* = 0.05).

	*E. alsinastrum*	*E. brochonii*	*E. campylosperma*	*E. gussonei*	*E. hexandra*	*E. hungarica*	*E. hydropiper*	*E. macropoda*	*E. orthosperma*	*E. triandra*
*E. alsinastrum*		abcdf	abcdef	acdef	abcef	abcde	cdef	abcde	aef	abcde
*E. brochonii*	abcdf		abcdef	abcdef	bcdef	cdef	abdef	abcdef	abcdef	acef
*E. campylosperma*	abcdef	abcdef		abcef	acdef	abdef	abc	cdef	bcde	abdef
*E. gussonei*	acdef	abcdef	abcef		abde	abcdf	ef	abcde	acdef	abcde
*E. hexandra*	abcef	bcdef	acdef	abde		bcdef	abcdef	ad	abcf	abcdf
*E. hungarica*	abcde	cdef	abdef	abcdf	bcdef		abcdef	abcdef	abcdef	abde
*E. hydropiper*	cdef	abdef	abc	ef	abcdef	abcdef		abcdef	acde	abcdef
*E. macropoda*	abcde	abcdef	cdef	abcde	ad	abcdef	abcdef		bcdef	abcde
*E. orthosperma*	aef	abcdef	bcde	acdef	abcf	abcdef	acde	bcdef		abcdf
*E. triandra*	abcde	acef	abdef	abcde	abcdf	abde	abcdef	abcde	abcdf	

**Notes.**

a surface b profile c rectangle a d rectangle b e angle of curvature f number of pits

**Figure 5:**
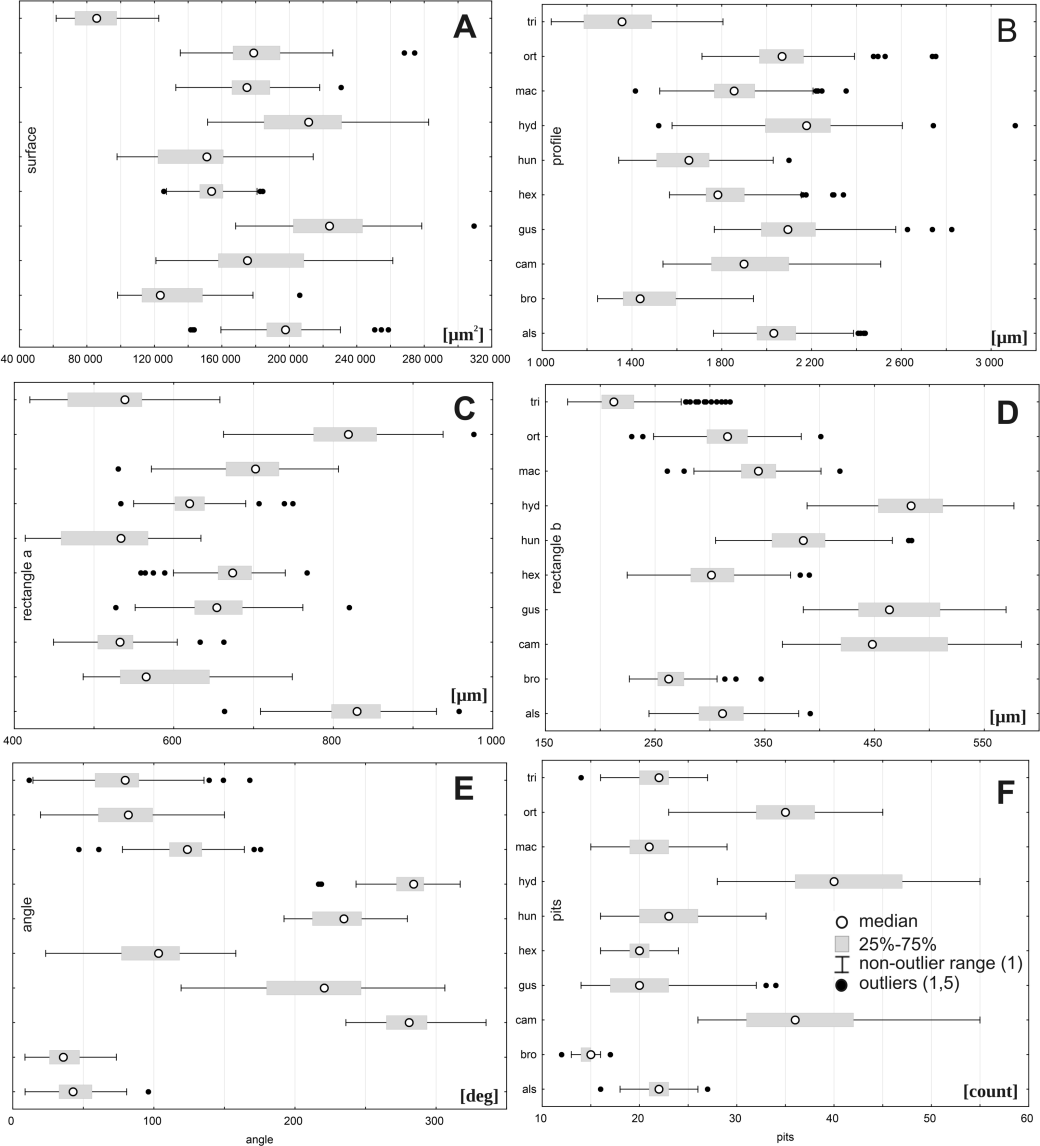
Boxplots of the most discriminative seed traits among *Elatine* species studied. Notations: boxes indicate 25–75 percentiles, white points indicate medians, whiskers exclude outliers, black points indicate outliers. For acronyms, see Table 1. (A) surface; (B) profile; (C) rectangle a; (D) rectangle b; (E) angle; (F) pits.

There was a large range of variation for the taxa studied regarding the following traits: seed size (traits: surface, profile, and rectangle a), especially within *E. hungarica* (SD 27183.7, SD 285.9, and SD 62.0, respectively); the angle of curvature, *E. gussonei* (SD 44.7); and number of pits, *E. campylosperma* (SD 7.2). The smallest variation was present in *E. triandra* (surface SD 14587.3, profile: SD 171.8, rectangle a: SD 54.6) and *E. brochonii* (rectangle b SD 20.6, the angle of curvature SD 14.7, pits: SD 1.3). The characteristics associated with size (surface, profile, rectangle a, rectangle b) revealed that the following species had the smallest seeds: *E. brochonii* and *E. triandra*, while the largest seeds in the studied species belonged to *E. gussonei* and *E. hydropiper* (Fig. 5, Table 7).

**Figure 6:**
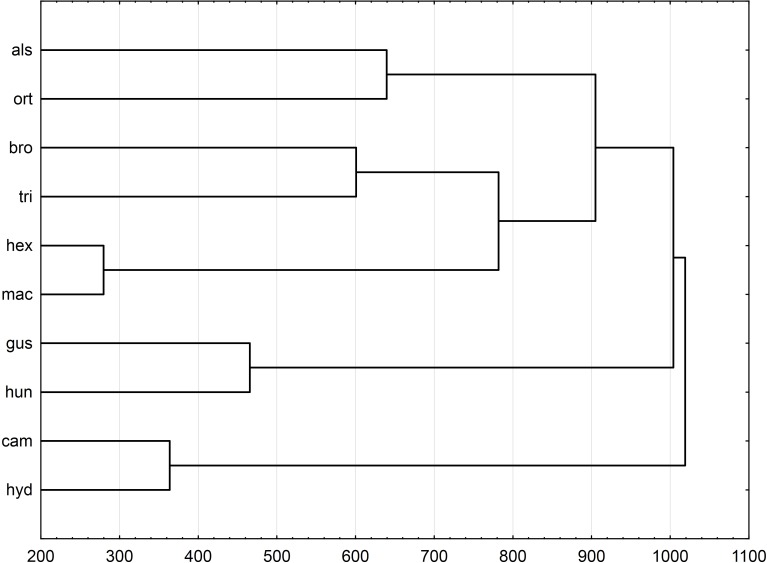
Morphological relationships of seeds among surveyed *Elatine* species displayed by Mahalanobis distance-based UPGMA cluster based on the following features: rectangle a, angle of curvature, and number of pits. For acronyms, see Table 1.

**Table 7:** SD values for seeds traits of the European *Elatine* species.

	Surface (µm^2^)	Profile (µm)	Rectangle_a (µm)	Rectangle_b (µm)	Angle of curvature (°)	Number of pits
*E. alsinastrum*	20213.4	142.3	50.1	27.8	16.7	1.8
*E. brochonii*	20881.3	133.8	58.9	20.6	14.7	1.3
*E. campylosperma*	30981.9	219.0	35.1	56.7	20.8	7.2
*E. gussonei*	26873.7	184.4	44.7	46.1	44.7	4.5
*E. hexandra*	11356.3	165.5	37.1	30.6	26.0	1.6
*E. hungarica*	27183.7	285.9	62.0	35.7	20.3	3.7
E. hydropiper	31653.4	250.3	33.6	41.5	17.3	6.6
*E. macropoda*	19610.4	148.5	50.3	30.6	20.9	2.5
*E. orthosperma*	22819.4	239.2	60.9	31.3	26.2	4.2
*E. triandra*	14587.3	171.8	54.6	32.2	27.5	2.7

The classification matrix of the discriminant analysis showed that the level of classification varied from 86% (rectangle a, the angle of curvature, number of pits) to 84% (surface, profile, rectangle a, rectangle b, the angle of curvature, number of pits). The highest values of classification were found for *E. alsinastrum, E, brochonii, E. hydropiper,* and *E. orthosperma* (all greater than 90%). The lowest values were found for *E. campylosperma* (57%, 55%) (Table 8).

**Table 8:** Classification matrix based on discriminant function analysis of seeds traits of *Elatine* species.

		*E. alsinastrum*	*E. brochonii*	*E. campylosperma*	*E. gussonei*	*E. hexandra*	*E. hungarica*	*E. hydropiperer*	*E. macropoda*	*E. orthosperma*	*E. triandra*	Correct *n*
Correct classification (%)	A	96	94	57	85	79	85	94	83	95	88	86
	B	97	92	55	78	71	82	95	78	93	91	84
*E. alsinastrum*	A	144	1	0	0	3	0	0	2	0	0	150
	B	145	1	0	0	4	0	0	0	0	0	150
*E.brochonii*	A	1	87	0	0	5	0	0	0	0	0	93
	B	1	86	0	0	5	0	0	0	0	1	93
*E. campylosperma*	A	0	0	55	0	0	17	25	0	0	0	97
	B	0	0	53	0	0	16	28	0	0	0	97
*E. gussonei*	A	0	0	1	126	0	1	4	16	0	0	148
	B	0	0	1	115	0	3	6	23	0	0	148
*E. hexandra*	A	1	1	0	0	105	0	0	26	0	0	133
	B	1	2	0	0	95	0	0	35	0	0	133
*E. hungarica*	A	0	0	1	16	0	99	0	0	0	0	116
	B	0	0	1	20	0	95	0	0	0	0	116
*E. hydropier*	A	0	0	6	2	0	0	119	0	0	0	127
	B	0	0	5	1	0	0	121	0	0	0	127
*E. macropoda*	A	5	0	0	0	17	0	0	115	0	1	138
	B	5	0	0	1	24	0	0	107	0	1	138
*E. orthosperma*	A	4	0	0	0	1	0	0	1	114	0	120
	B	6	0	0	0	1	0	0	1	112	0	120
*E. triandra*	A	0	1	0	0	15	1	0	0	0	121	138
	B	0	1	0	0	9	1	0	2	0	125	138
Total classified	A	155	90	63	144	146	118	148	160	114	122	1,260
	B	158	90	60	137	138	115	155	168	112	127	1,260
(Correct *n*) − (Tot. class.)	A	**−5**	**3**	**34**	**4**	**−13**	**−2**	**−21**	**−22**	**6**	**16**	
	B	**−8**	**3**	**37**	**11**	**−5**	**1**	**−28**	**−30**	**8**	**11**	

**Notes.**

A rectangle a, angle of curvature, pits B surface, profile, rectangle a, rectangle b, angle of curvature, number of pits

**Figure 7:**
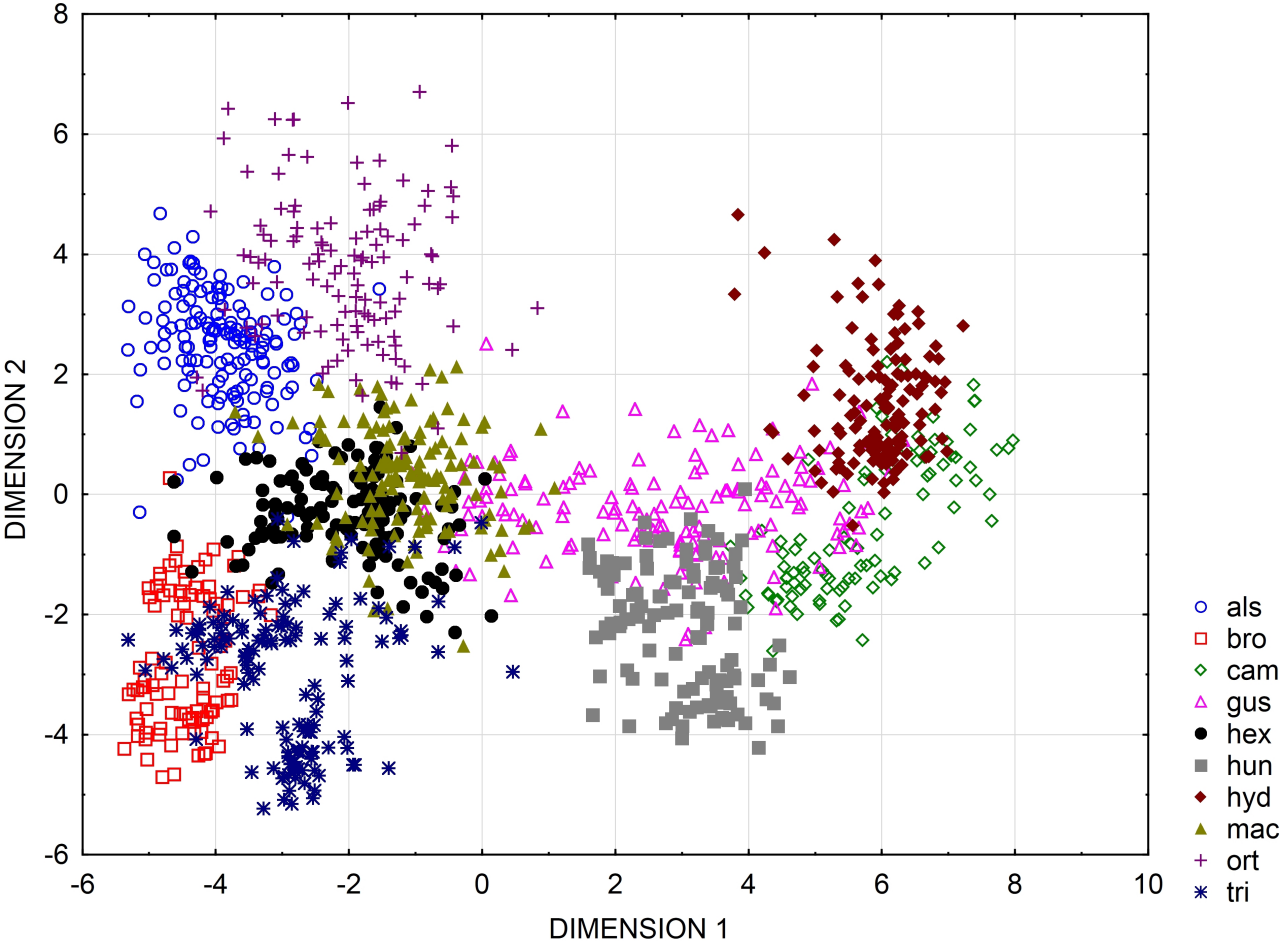
Categorized scatterplot based on canonical analysis value for seeds of the European species of *Elatine*. For acronyms, see Table 1.

UPGMA clusters of Mahalanobis distance based on rectangle a, the angle of curvature, and number of pits yielded two groups: species with straight or nearly straight seeds, and species with curved and U-shaped seeds (Fig. 6). The greatest similarity was found between seeds of *E. hexandra* and *E. macropoda,* and *E. campylosperma* and *E. hydropiper.* The spatial distribution of observed characteristics of the analyzed species is depicted as a categorized scatterplot based on canonical analysis values (Fig. 7).

## Discussion

Our study shows that in *Elatine* tested seed variability is mainly associated with size-connected traits, especially surface, profile, rectangle b, and, to a lesser extent, rectangle a. This allowed us to draw the conclusion that to distinguish seeds of these species the most useful traits are the angle of curvature and number of pits, and to a lesser extent rectangle a (length). These findings confirm previous knowledge about the usefulness of these features in *Elatine* taxonomy (Misfud, 2006; Uotila, 2009a; Uotila, 2010; Molnár et al., 2013; Molnár et al., 2015). Nevertheless, our study revealed that the range of variation of European *Elatine* morphological features is large, both between species and the populations of each species.

Regarding intraspecific variability, the traits studied were not statistically significantly different between studied populations of the following taxa: *E. alsinastrum, E. macropoda, E. hexandra.* Conversely, *E. gussonei, E. campylosperma E. hungarica* and *E. hydropiper* seeds showed statistically significant intrapopulation variability. The taxonomic status of the first three species is still being elucidated. *Elatine gussonei*, an enigmatic plant of the Mediterranean, was first described as a variety of *E. hydropiper* and was later classified as a separate species (Brullo et al., 1988; Misfud, 2006; Molnár et al., 2013; Molnár, Popiela & Lukács, 2013). *Elatine campylosperma* was described by Seubert (1845) from Sardinia, and later greatly neglected by most researchers by synonymizing this species under *E. macropoda*; at present, it is considered a separate species (Kalinka et al., 2015). *Elatine hungarica* was last collected in 1960 and rediscovered in Hungary in 1998 (Molnár et al., 1999); for years its taxonomic status was under discussion (Molnár et al., 2013).

Our present study showed that regarding shape statistically only *E. alsinastrum* and *E. orthosperma* seeds are nearly straight and seeds of all other species are curved to varying degrees; the range of variation in some species is large in this respect, especially in *E. gussonei, E. triandra*, and *E. hexandra*.

**Figure 8:**
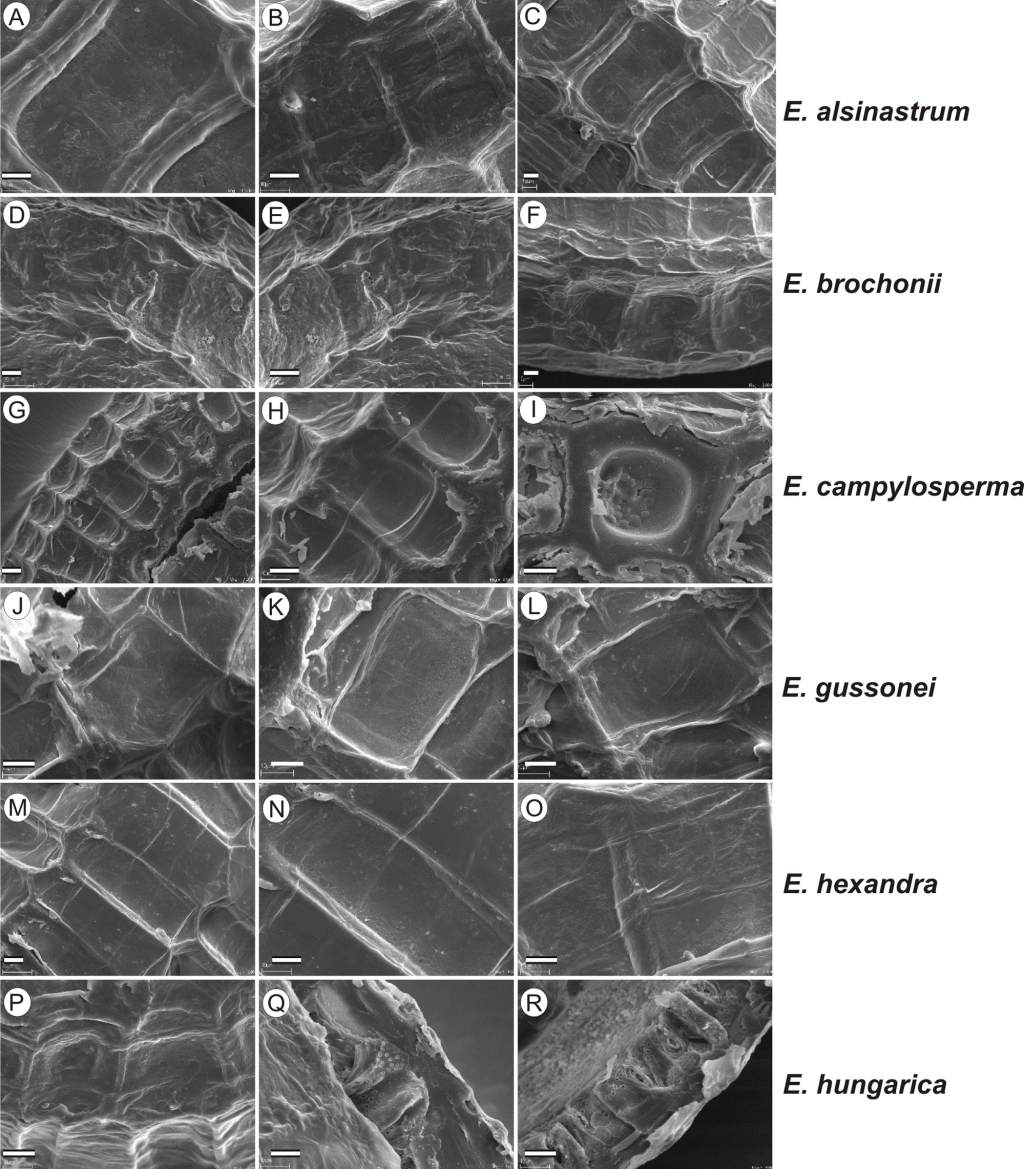
The diversity in seed coat micromorphology of *Elatine alsinastrum* (a–alsHu; b, c–alsPL1), *E. brochonii* (a, b–broMO; c–broSP), *E. campylosperma* (a, b–camIT; c–camSP), *E. gussonei* (a, b–gusMAL; c–gusSP), *E. hexandra* (a, b–hexPL1; c–hexPL2), *E. hungarica* (a, b–hunR; c–hunSL). Scale bar = 10 µm. For acronyms, see Table 1. (A–C) *E. alsinastrum*; (D–F) *E. brochonii*; (G–I) *E. campylosperma*; (J–L) *E. gussonei*; (M–O) *E. hexandra*; (P–R) *E. hungarica*.

**Figure 9:**
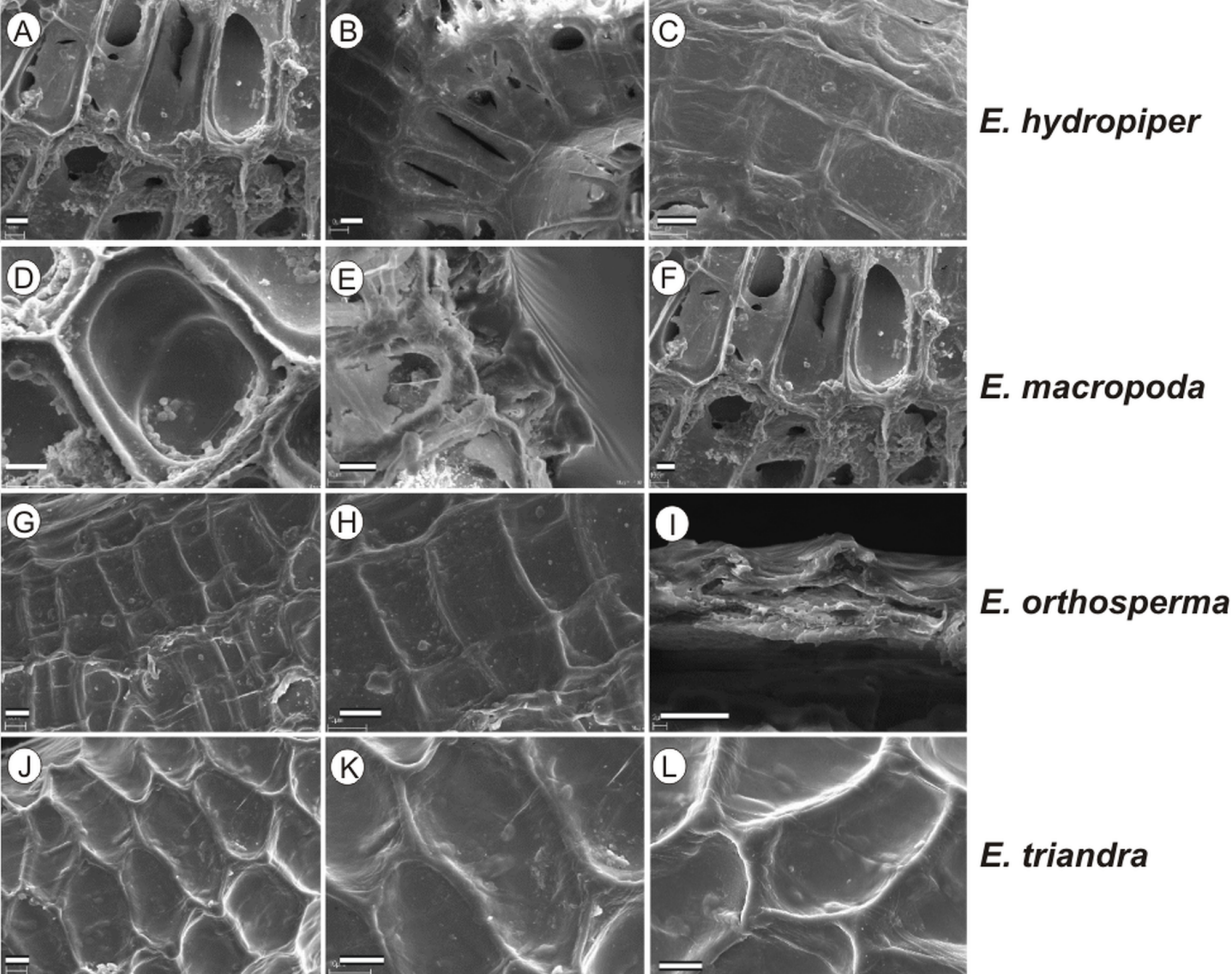
The diversity in seed coat micromorphology of *Elatine hydropiper* (a - hydHu, b, c - hydPL1); *E. macropoda* (a, b -macIT; c–macSP), *E. orthosperma* (a, b -ortCZ; c - ortFI1), *E. triandra* (a–triHU; b, c–triPL1). Scale bar = 10 µm. For acronyms, see Table 1. (A–B) *E. hydropiper*; (D–F) *E. macropoda*; (G–I) *E. orthosperma*; (J–L) *E. triandra*.

**Figure 10:**
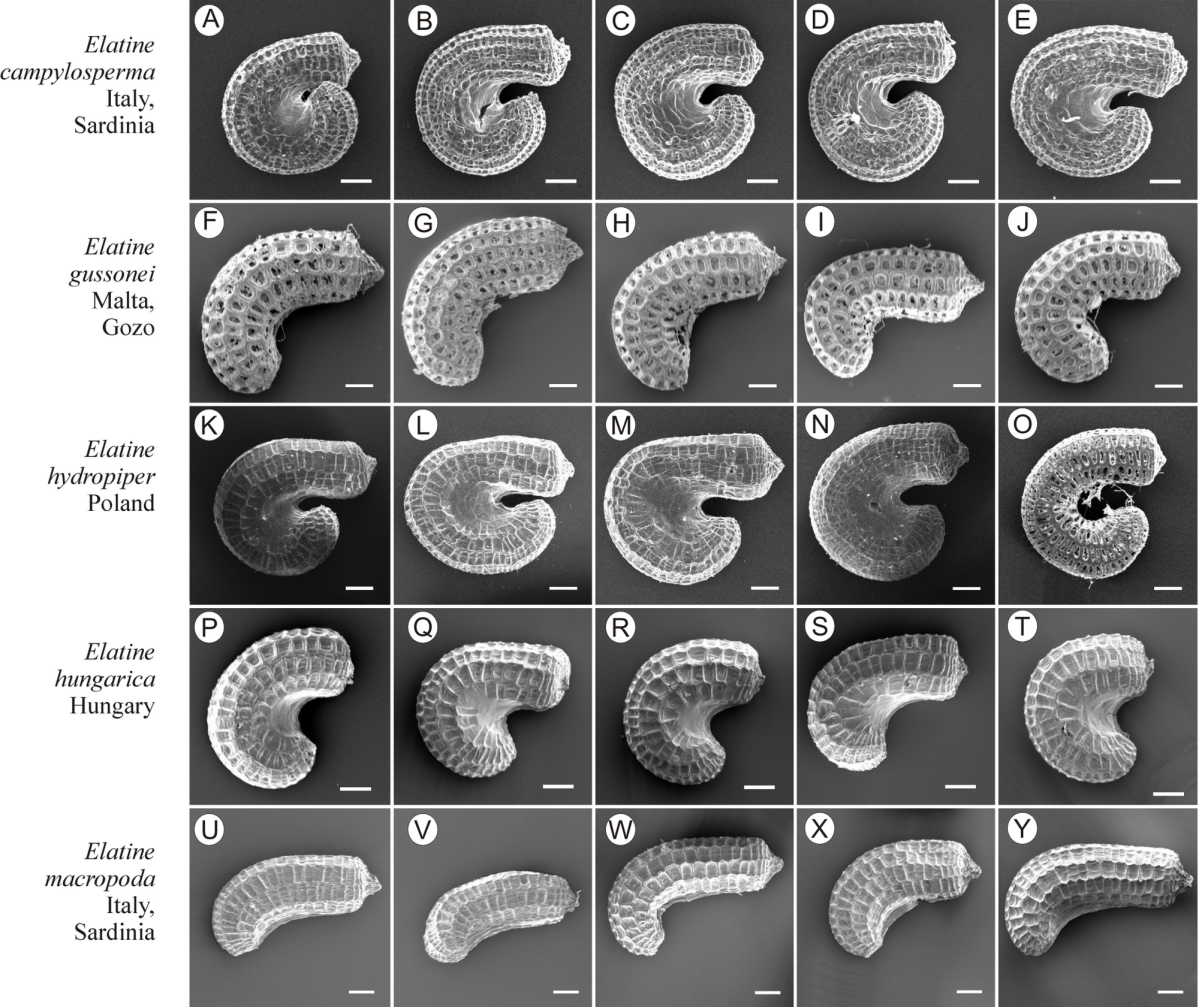
The diversity of seeds of *Elatine campylosperma, E. gussonei, E. hydropiper, E. hungarica, E. macropoda*. Scale bar =200 µm. (A–E) *E. campylosperma*; (F–J) *E. gussonei*; (K–O) *E. hydropiper*; (P–T) *E. hungarica*; (U–Y) *E. macropoda*.

**Figure 11:**
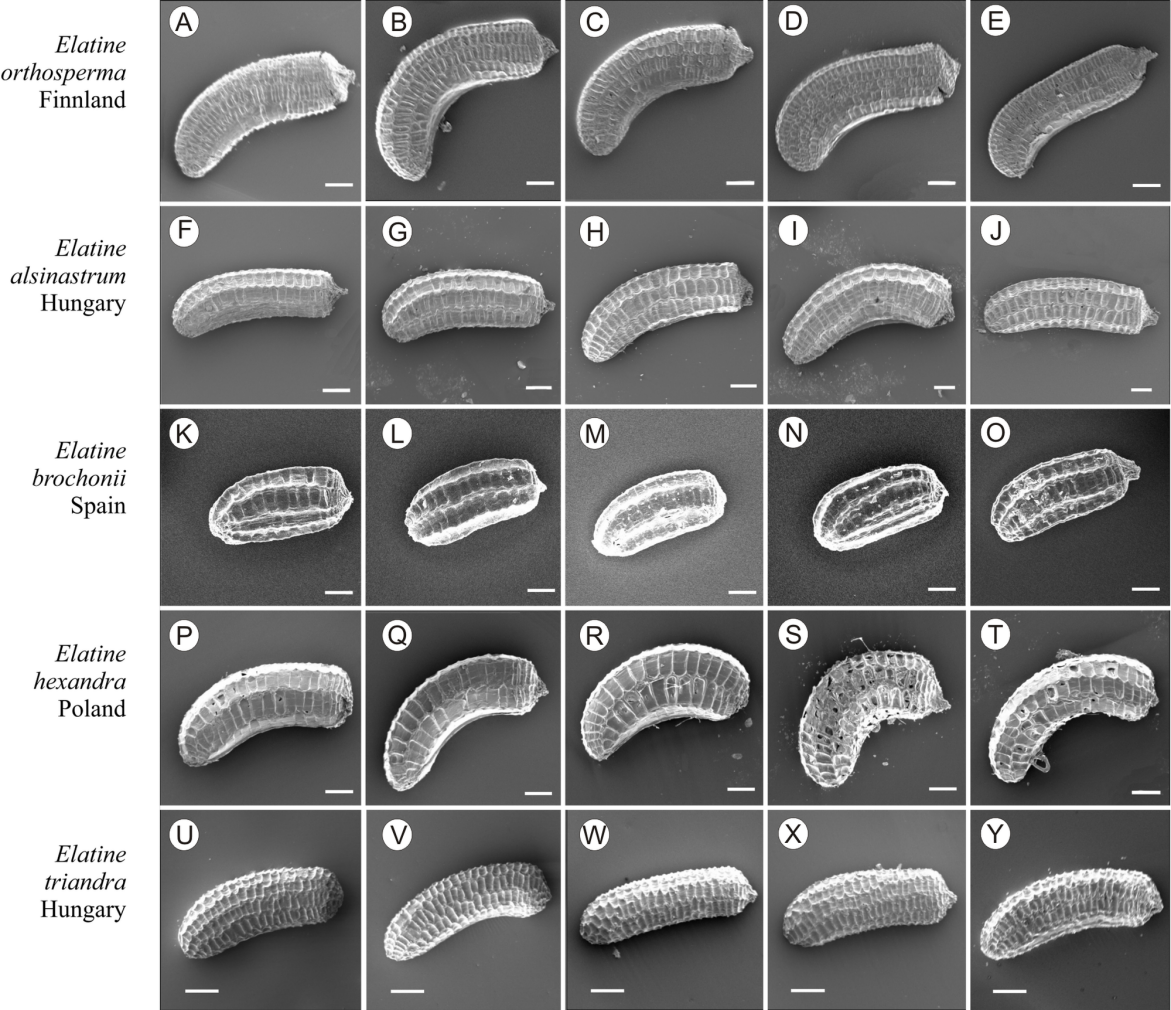
The diversity of seeds of *Elatine orthosperma, E. alsinastrum, E. brochonii, E. hexandra, E. triandra*. Scale bar =200 µm. (A–E) *E. orthosperma*; (F–J) *E. alsinastrum*; (K–O) *E. brochonii*; (P–T) *E. hexandra*; (U–Y) *E. triandra*.

If distinction of species is only based on seeds, it would be easy to confuse the following species pairs: *E. alsinastrum* and *E. orthosperma*, *E. hexandra* and *E. macropoda*, *E. campylosperma* and *E. hydropiper,* and*E. gussonei* and *E. hungarica,* especially if only a few seeds are evaluated. Previously, Misfud (2006), who worked on Malta and Mallorca populations, pointed out the importance of distinction based on greater seed curvature in *E. gussonei* compared with *E. macropoda*; although this is true if averages are used, there is a substantial amount of overlap in curvature and this could lead to confusion if the curvature of only few seeds are analyzed. This was also confirmed by our results. Misfud (2006) also drew attention to the distinctive seed testa reticulation, and claimed that the wide hexagonal shape of pits in *E. gussonei* and smaller number of pits/row (15 ± 3) are very difficult to confuse with *E. macropoda*’s 21 ± 3 narrow pits/row. Our study yielded different results: seeds of *E. macropoda* populations had similar number of pits [(13–)19–23–(29)] compared to *E. gussonei* [(17–)23(–32)]. However, because Misfud (2006) did not precisely describe the method of counting pits (especially in which row pits were counted), it is difficult to compare our results. Molnár et al. (2013) pointed out that seeds of *E. hungarica* are much more curved than those of *E. gussonei*, and especially of *E. macropoda* and *E. orthosperma*, but somewhat less curved than those of *E. hydropiper*. Our current study revealed that the range of variation for the feature the angle of curvature of *E. hungarica* seeds is similar to that of *E. gussonei*, and more curved seeds are found in *E. hydropiper* and *E. campylosperma*. These results are basically consistent with observations of Molnár et al. (2013), especially considering that more varied material was used in the current study. Our research confirms observations of Misfud (2006) and Molnár et al. (2013) concerning the evident semilunar membrane on the concave side of seeds (Figs. 8–11). The membrane was present and clearly visible in all highly curved fresh seeds of the following species: *E. gussonei, E. hydropiper, E. hungarica*, and *E. campylosperma*. Regarding the seed testa, a very distinctive network-shape ornamentation pattern of *E. triandra* as visible (Figs. 9J– 9L, 11). *Elatine campylosperma* seeds showed the most distinctive reticulation, and were characterized by a large number of narrow, rectangular pits (Fig. 10), and round-shaped pits (Figs. 8G– 8I). Similarly, rectangular-shaped pits were found in seeds of the following species: *E. alsinastrum*, *E. triandra, E. orthosperma, E. hydropiper,* and *E. macropoda*; however, hexagonal pits were also present (Figs. 10 and 11). The pit shapes of *E. gussonei, E. hungarica, E. brochonii*, and *E. hexandra* are usually both hexagonal and rectangular, with a predominance of the former (Figs. 10 and 11). Similar observations for some of these species were made by Misfud (2006), Molnár et al. (2013), Molnár, Popiela & Lukács (2013). We believe that the shape of pits may be an additional feature that helps distinguish seeds of individual species (Figs. 8 and 10). However, we found no diversity in seed coat micromorphology within pits (e.g., pores, strophioles) that could have potential taxonomic importance. Seed coats within pits were smooth with the exception of irregular strips, and the porosity of the seed coat is visible only in the inner layer of cracked seeds (Figs. 8Q–8R, 9I). Ornamentation pattern (pit shape) becomes distinct as the seeds dry up. The outer layer of the seed coat is very thin and easily destroyed ( Figs. 9A–9B; 9D–9F).

Our research allowed us to construct a guide that can be useful to identify the studied taxa based on seed traits. We believe that this guide is important for better recognition of these rare and endangered species, and can be useful for elucidating the history of range formation of these taxa in the Holocene and their origin. * Elatine* subfossil finds were discovered in late-glacial and pre-boreal sediments in the last few centuries (Latałowa, 1992; Brinkkemper et al., 2008; Kowalewski et al., 2013). The ecological amplitude of this species provides robust clues for environmental reconstruction, which must have been a temporarily flooded fresh water area. “...since this type of environment is strongly threatened on a worldwide scale, the presence of these species in the past may also provide interesting information for present nature development projects...” (Brinkkemper et al., 2008).

Identification guide and descriptions for European species of *Elatine* based on seed morphology presented in Figs. 10 and 11. Note: the guide does not include exceptional values given in parentheses in the descriptions (min. outliers 1.5) 25%–75% (max. outliers 1.5).

**Table:** 

**1** Seeds straight –almost straight –slightly curved, the angle of curvature < 150°	**2**
1* Seeds curved or U-shaped, the angle of curvature ≥ 150	**8**
**2** Number of pits in the seed coat in the middle row ≥ 30	*E. orthosperma*

Seed length (658–)776–854(–971) µm, width (242–)297–334(–389) µm, angle of curvature (55–)61–99(–156)° number of pits in the middle row (23–)32–38(–47), prevailing pit shape rectangular, semilunar membrane absent on the concave side of seeds.

**Table:** 

2* Number of pits in the seed coat in the middle row <30	**3**
**3** Length of seeds < 600 µm	**4**
3* Length of seeds ≥ 600 µm	**5**
**4** Number of pits in the seed coat in the middle row < 17	*E. brochonii*

Seed length (365–)533–645(–813) µm, width (217–)252–276(–312) µm, angle of curvature: 26–47(–79)°, number of pits in the middle row (12–)14–15(–17), prevailing pit shape hexangular, semilunar membrane absent on the concave side of seeds.

**Table:** 

4* Number of pits in the seed coat in the middle row ≥ 18	*E. triandra*

Seed length (328)467–560(700) µm, width: (158)201–231(274) µm; angle of curvature: 58–89 (136)°, number of pits in the middle row (16)20–23(28), prevailing network-shape of pits in the seed coat, semilunar membrane absent on the concave side of seeds.

**Table:** 

**5** Number of pits in the seed coat in the middle row ≤17	*E. brochonii* (for description see above, after line **4**)
5* Number of pits in the seed coat in the middle row >17	**6**
**6** Angle of curvature of seeds ≤60°	*E. alsinastrum*

Seed length (708–)799–859(–950) µm, width: (230)290–330(391) µm, angle of curvature: 33–56(91)°, number of pits in the middle row: (18)21–23(26), prevailing rectangular shape of pits in the seed coat, semilunar membrane absent on the concave side of seeds.

**Table:** 

6*Angle of curvature of seeds > 60°	**7**
**7** Width of seeds ≥ 320 µm	*E. macropoda*

Seed length: (568–)666–732(–830) µm, width: (282–)329–360(–407) µm, angle of curvature (78–)111–134(–167)°, number of pits in the middle row: (13–)19–23(–29); prevailing rectangular shape of pits in the seed coat, usually semilunar membrane absent on the concave side of seeds.

**Table:** 

7* Width of seeds < 320 µm	*E. hexandra*

Seed length: (593–)656–697(–760) µm, width: (223–)283–322(–381) µm; angle of curvation (15–) 77–118(–180)°, number of pits in the middle row: (16–)19–21(–24), the shape of pits in the seed coat hexagonal and rectangular, semilunar membrane absent on the concave side of seeds.

**Table:** 

**8** Number of pits in the seed coat in the middle row ≥ 30	**9**
8* Number of pits in the seed coat in the middle row <30	**10**
**9** Length of seeds < 600 µm	*E. campylosperma*

Seed length: (439–)505–549(–615) µm, width: (274–)419–517(–663) µm, angle of curvation: (222–)265–294(–337)°, number of pits in the middle row: (15–)31–42(–59), narrow rectangular or round shape of pits in the seed coat, semilunar membrane present on the concave side of the seeds.

**Table:** 

9* Length of seeds ≥600 µm	*E.hydropiper*

Seed length: (548–)602–638(–693) µm, width: (367–)454–512(–599) µm, angle of curvation: (246–)273–291(–318)°, number of pits in the middle row: (22–)37–48(–62), prevailing rectangular shape of pits in the seed coat, semilunar membrane present on the concave side of the seeds.

**Table:** 

**10** Length of seeds ≤ 600 µm	*E. hungarica*

Seed length: (296–)459–567(–730) µm, width: (284–)357–405(–477) µm; angle of curvation: (161–)213–247(–299)°, number of pits in the middle row: (11–)20–26(–35), prevailing hexagonal shape of pits in the seed coat, semilunar membrane present on the concave side of the seeds.

**Table:** 

10* Length of seeds > 600 µm	*E. gussonei*

Seed length: (539–)627–685(–774) µm, width: (325–)436–509(–620) µm; angle of curvation: (80–)180–247(–347)°, number of pits in the middle row: 17–23(–32), prevailing hexagonal shape of pits in the seed coat, semilunar membrane present on the concave side of the seeds.

##  Supplemental Information

10.7717/peerj.3399/supp-1 Supplemental Information 1 Source data

## Additional Information and Declarations

### Field Study Permissions

The following information was supplied relating to field study approvals (i.e., approving body and any reference numbers): *Elatine hungarica*, *E. hydropiper* and *E. triandra* are protected species and were sampled in Hungary with the permission of the Hortobágy National Park Directorate (Permission id.: 45-2/2000, 250-2/2001).

### Data Availability

The following information was supplied regarding data availability: The raw data has been supplied as Supplemental Information 1.
